# Comment on Barbu et al. Can Thrombosed Abdominal Aortic Dissecting Aneurysm Cause Mesenteric Artery Thrombosis and Ischemic Colitis?—A Case Report and a Review of Literature. *J. Clin. Med.* 2025, *14*, 3092

**DOI:** 10.3390/jcm14134672

**Published:** 2025-07-02

**Authors:** Sheng-Nan Wu, Chung-Hung Tsai

**Affiliations:** 1 Department of Research and Education, An Nan Hospital, China Medical University, Tainan 70965, Taiwan; 2 Institute of Allied Health Science, College of Medicine, National Cheng Kung University, Tainan 70101, Taiwan; chunghong.kuanyin@gmail.com; 3 Department of Family Medicine, An Nan Hospital, China Medical University, Tainan 70965, Taiwan

We recently came across an interesting article published in your journal, titled “Can Thrombosed Abdominal Aortic Dissecting Aneurysm Cause Mesenteric Artery Thrombosis and Ischemic Colitis?—A Case Report and a Review of Literature” by Dr. Barbu et al., published in *Journal of Clinical Medicine* 2025;14:3092 [1].

This case is indeed noteworthy. However, we would like to raise five notable points that we believe merit further clarification:

1. The article provides both detailed CT imaging and operative reports for the patient but does not include a discussion of the physical examination findings for the upper and lower limbs [1]. Crucial evaluations, such as toe pule measurements (Figure 1), Doppler studies, and palpation findings, are essential for the early detection of possible peripheral artery diseases. This disease includes abdominal aortic aneurysms and aortic coarctation. These assessments, especially when compared to upper limb findings (as illustrated in Figure 2), provide valuable diagnostic insights. Unfortunately, such physical examination methods are often overlooked in clinical practice [1,2,3,4,5,6]. Additionally, Figure 3 shown here displays a real-time graph of vital signs, including ECG, pulse, and respiratory rate, captured digitally using a Philips^®^ Patient Monitor.
2. Pulse measurement is particularly important in patients with peripheral arterial disease, as they are at increased risk of sudden death. This is because inadequate physical examination may lead to inappropriate cardiopulmonary resuscitation, which could potentially cause a fatal aneurysmal rupture during chest compressions (i.e., cardiac massage).
3. Regarding the etiology of abdominal aortic aneurysm, the article mentions that the cause of abdominal aortic aneurysm remains unclear [1]. An abdominal aneurysm refers to an abnormal, localized dilation of bulging of the abdominal aorta, the main artery that supplies blood to the lower part of the body. However, previous studies have indicated a strong association with long-standing uncontrolled hypertension, as reported previously [7,8,9]. We would appreciate the authors’ further elaboration on this point. Additionally, it is unclear whether the patient had a history of syphilis or any hereditary conditions such as Marfan syndrome. Patients with syphilis may develop syphilitic aortitis, which can lead to the formation of aortic aneurysm. Syphilitic aortitis occurs due to the chronic inflammatory response triggered by Treponema pallidum. The bacterium causes damage to the aortic wall, leading to the inflammation, thickening, and weakening of the aortic tissues [10]. Syphilitic aortitis is more likely, because it occurs in the tertiary stage, 30 years after diagnosis, but it is also rare, especially if a course of treatment with penicillin was administered, for which (or risk factors) there is no information in the patient’s personal history.
4. Marfan syndrome is a genetic disorder that affects the connective tissue in the body, which provides structure and support to organs, blood vessels, bones, and tissues. The most serious complications of Marfan syndrome often involve the heart and blood vessels, particularly the aorta. Aortic aneurysm may occur, potentially leading to aortic dissection, which is life-threatening [11]. In addition, these patients have a characteristic phenotype [11,12]: they are tall and slim, with eye changes (cornea and/or lens), so they rarely go undiagnosed.
5. It is important to note that Marfan syndrome is typically diagnosed early in life [12], making it unlikely that the condition significantly contributed to vascular changes in a patient of advanced age [1]. We agree that the diagnostic algorithm for the patient should have been presented more clearly and thoroughly, in which all differential–diagnostic and etiological dilemmas would be resolved [1]. A long-term patient with numerous comorbidities certainly has a resolution regarding the etiology of the mentioned diseases, which culminated in thrombosis and ischemia. The patient’s history of comorbidities clearly indicates that the patient is a long-term cardiology patient who has undergone all diagnostic evaluations [1]. Moreover, the possibility of other genetic and non-genetic factors influencing vascular pathology in elderly patients needs to be investigated.

## Figures and Tables

**Figure 1:**
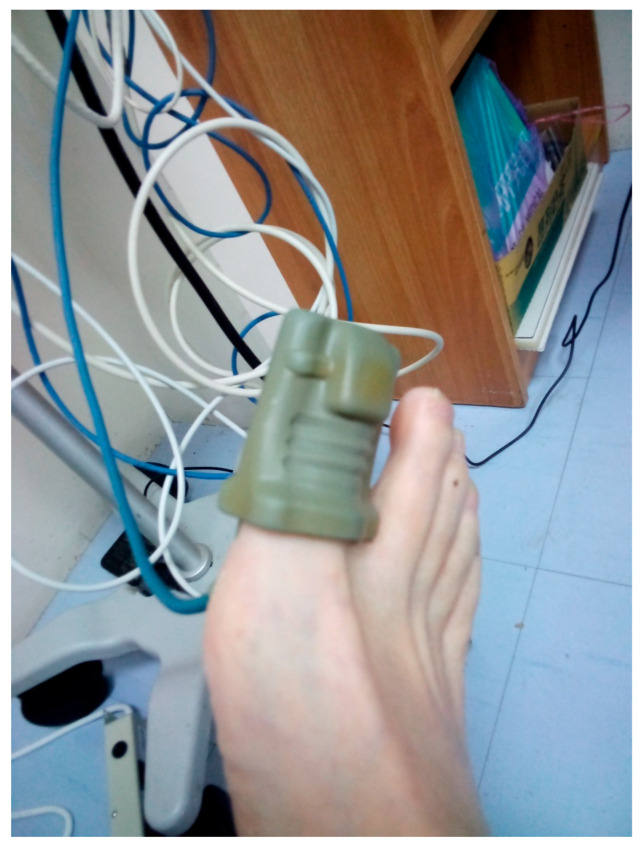
Measurement at the toe using a pulse biosensor (with a dark green sensor head). This is one of the important maneuvers for assessing peripheral arterials diseases, such as the coarctation of the aorta or dissecting aortic aneurysm.

**Figure 2:**
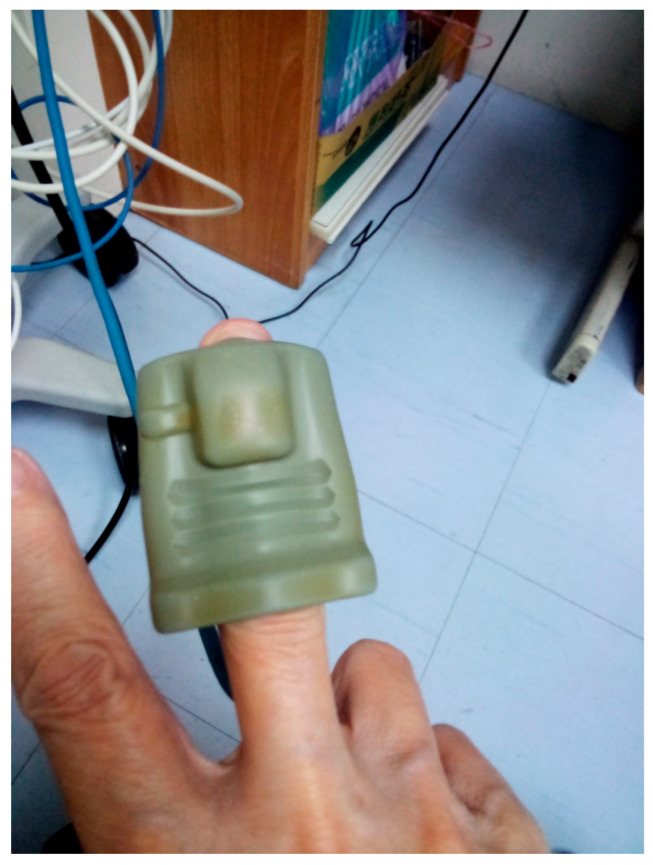
Pulse measurement at the index finger of the upper limb. Both of the above measurements are connected to the Philips^®^ IntelliVue Patient Monitor, allowing for real-time detection of digitized pulse signals. Importantly, assessing and comparing pulse measurements from both the upper and lower limbs in the same patient can aid in the early detection and management of various peripheral arterial diseases.

**Figure 3:**
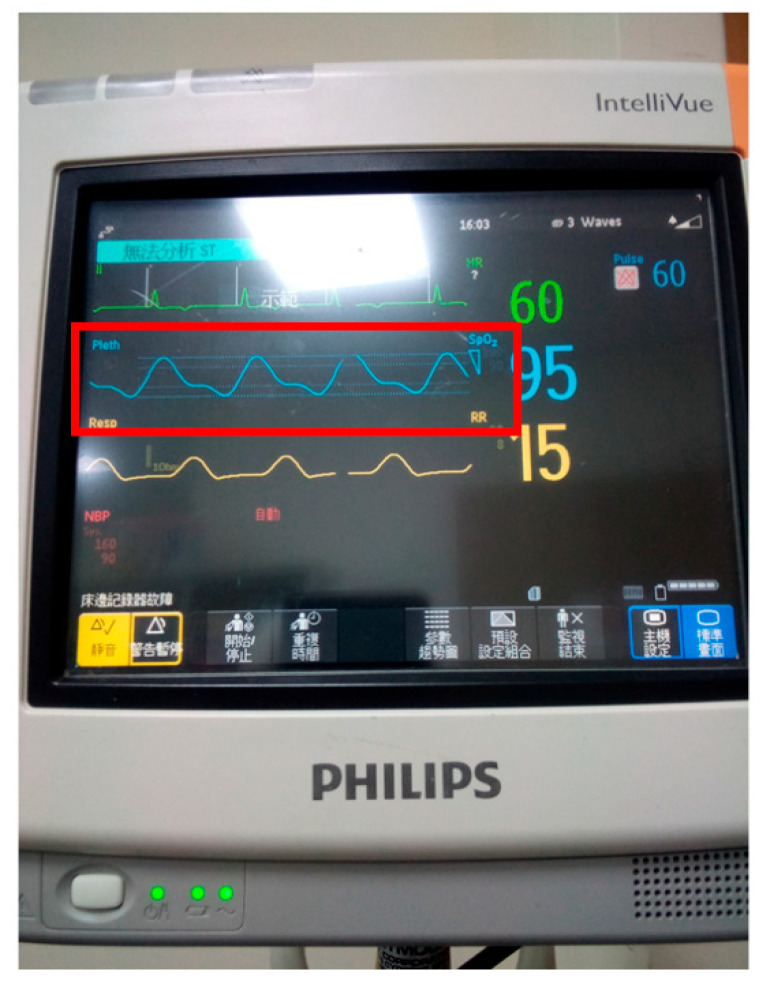
The real-time monitoring of digitized vital signs, including ECG, finger pulse and respiratory rate, captured using the Philip^®^ IntelliVue MP5 monitor (MediGiant Enterprise Co., Ltd., Kaohsiung, Taiwan). The extended red horizontal box marked in the graph highlights the beat-to-beat progression of the finger pulse waveform. Of note, under normal physiological conditions, pulse waveforms measured from the upper and lower limbs, such as finger and toe pulses, are expected to exhibit similar patterns in both shape and amplitude.

## Data Availability

Data are available on request.
